# FGF23 Deficiency Leads to Mixed Hearing Loss and Middle Ear Malformation in Mice

**DOI:** 10.1371/journal.pone.0107681

**Published:** 2014-09-22

**Authors:** Andrew C. Lysaght, Quan Yuan, Yi Fan, Neil Kalwani, Paul Caruso, MaryBeth Cunnane, Beate Lanske, Konstantina M. Stanković

**Affiliations:** 1 Program in Speech and Hearing Bioscience and Technology, Harvard/MIT Joint Division of Health Sciences and Technology, Cambridge, Massachusetts, United States of America; 2 Eaton Peabody Laboratories, Massachusetts Eye and Ear Infirmary, Boston, Massachusetts, United States of America; 3 Department of Oral Medicine, Infection, and Immunity, Harvard School of Dental Medicine, Boston, Massachusetts, United States of America; 4 Department of Radiology, Massachusetts Eye and Ear Infirmary and Harvard Medical School, Boston, Massachusetts, United States of America; 5 Department of Otology and Laryngology, Harvard Medical School, Boston, Massachusetts, United States of America; 6 Department of Otolaryngology, Massachusetts Eye and Ear Infirmary, Boston, Massachusetts, United States of America; Universitat Pompeu Fabra, Spain

## Abstract

Fibroblast growth factor 23 (FGF23) is a circulating hormone important in phosphate homeostasis. Abnormal serum levels of FGF23 result in systemic pathologies in humans and mice, including renal phosphate wasting diseases and hyperphosphatemia. We sought to uncover the role FGF23 plays in the auditory system due to shared molecular mechanisms and genetic pathways between ear and kidney development, the critical roles multiple FGFs play in auditory development and the known hearing phenotype in mice deficient in klotho (KL), a critical co-factor for FGF23 signaling. Using functional assessments of hearing, we demonstrate that *Fgf* 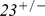 mice are profoundly deaf. *Fgf* 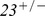 mice have moderate hearing loss above 20 kHz, consistent with mixed conductive and sensorineural pathology of both middle and inner ear origin. Histology and high-voltage X-ray computed tomography of *Fgf* 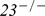 mice demonstrate dysplastic bulla and ossicles; *Fgf* 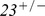 mice have near-normal morphology. The cochleae of mutant mice appear nearly normal on gross and microscopic inspection. In wild type mice, FGF23 is ubiquitously expressed throughout the cochlea. Measurements from *Fgf* 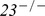 mice do not match the auditory phenotype of *Kl* ^−/−^ mice, suggesting that loss of FGF23 activity impacts the auditory system via mechanisms at least partially independent of KL. Given the extensive middle ear malformations and the overlap of initiation of FGF23 activity and Eustachian tube development, this work suggests a possible role for FGF23 in otitis media.

## Introduction

Fibroblast growth factor 23 (FGF23) is a circulating hormone, typically secreted by osteoblasts and osteocytes, which regulates renal phosphate handling [1], [2], [3]. Phosphate wasting diseases (including tumor-induced osteomalacia, X-linked and autosomal-dominant hypophosphatemic rickets, all of which may present with hearing phenotypes) occur with elevated serum levels of FGF23 [4], [5], [6]. Reduced FGF23 activity leads to decreased urinary excretion of phosphate [7], as in familial tumoral calcinosis. Mice deficient in *Fgf* 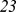 exhibit severe hyperphosphatemia, hypervitaminosis D, hypercalcemia, ectopic calcifications, growth retardation, organ atrophy, infertility and shortened life expectancy [8], [9], [10], [2].

This study sought to characterize the murine auditory phenotype due to FGF23 deficiency because other members of the FGF superfamily are critical for normal development of the auditory system in humans and animal models [11], [12], [13], [14], kidney and inner ear development rely on shared molecular mechanisms and genetic pathways [15], [16] and both organs are susceptible to toxicity from aminoglycoside antibiotics and loop diuretics [16], [17], [18]. Additionally, deficiency in klotho (KL), a critical co-factor for FGF23 mediated signaling [19], [20], results in hearing loss [21], [22]. Alpha-KL is a single-pass transmembrane protein that increases FGF receptor (FGFR) affinity for FGF23 [19], [20] and decreases affinity to other FGFs. KL expression has been reported in several cochlear cell types [22], [23], but the sensory epithelia of *Kl* ^−/−^ mice appear normal [21]. The skeletal phenotypes present in *Fgf* 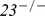 mice are also present in *Kl* ^−/−^ mice and double knockouts. Bioactive FGF23 injection results in phenotypic rescue of *Fgf* 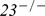 mice but not in KL knockouts [24]. A vitamin D-deficient diet rescues the auditory and systemic *Kl* ^−/−^ phenotypes [21], [25]. Injection of just the C-terminal tail of FGF23, capable of binding the FGFR1c-Klotho complex but not regulating phosphate handling, can reduce phosphate wasting via receptor competition in both normal and FGF23 over-expresser mice [26].

It is currently unclear if the *Kl* ^−/−^ auditory phenotype is due to altered FGF23 signaling because KL and FGF23 can exhibit functions independent of each other [27], [28]. However, the leading hypothesis is that hearing loss results from demineralization of the auditory ossicles, resulting from hypervitaminosis D [21] induced by loss of renal FGF23 activity. If correct, hearing loss should be seen in *Fgf* 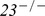 mice and should be conductive in nature. To understand the relevance of FGF23 in auditory function, we studied *Fgf* 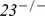 and *Fgf* 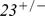 mice using physiologic, histologic and radiologic tools. We demonstrate that FGF23 is critical for normal development of the middle ear and for function of both the middle and inner ear. We also demonstrate a connection between FGF23 deficiency and predilection to otitis media. This finding has diagnostic and therapeutic implications as the genetic basis of human otitis media is poorly understood [29] and otitis media is the most common cause for pediatric antibiotic and surgical intervention [30].

## Materials and Methods

For this study, we utilized a previously developed *Fgf* 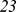 null mouse (*Fgf* 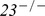) [2], [24], [31] in which the entire *Fgf* 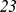 gene has been replaced with the *lacZ* gene. Male, 6-week old mice were generated via the breeding of *Fgf* 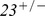 mice. Genotypes were confirmed with PCR. All studies were approved by the Institutional Animal Care and Use Committee at Harvard Medical School.

### Audiometric testing

Physiologic performance of the auditory systems of *Fgf* 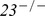, *Fgf* 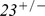, and *Fgf* 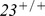 littermates was assessed using auditory brainstem response (ABR), which assess organ and neural function, and distortion product oto-acoustic emission (DPOAE) measurements, which assess middle ear function and cochlear amplification. ABR waveforms were recorded from anesthetized mice (ketamine 0.1 mg/g and xylazine 0.02 mg/g) via sub-dermal electrodes placed at the ipsilateral pinna and vertex (grounded at the junction of the tail and torso). A brief tone was presented into the ipsilateral ear canal and the resulting electrode waveform was recorded and averaged (512 presentations, presented at 40 Hz). Tones were presented at frequencies from 5.66–45.25 kHz in half-octave steps and intensities ranging 15–80 dB SPL in 5 dB steps. Threshold was defined as the first intensity at which recognizable and repeatable peaks became observable in the averaged recording. ABR wave I amplitude (measured peak-to-peak from the local maxima of 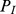 to the local minima of 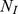) and latency were determined for each frequency and intensity using the ABR Peak Analysis software (Bradley Buran, Eaton-Peabody Laboratory). Amplitude and latency values were calculated for the middle 5 frequency points due to increased noise in the highest and lowest measurements. Latency values are presented in dB SL (sensation level), as opposed to dB SPL like amplitude measurements. At high enough stimulation intensities (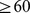 dB SPL tones) ABR amplitudes have a greater dependance on absolute intensity due to growth of excitation and cochlear saturation.

DPOAEs were measured in anesthetized mice via simultaneous presentation of two tones (denoted 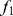 and 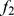, the frequency of 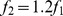 Hz and intensity of 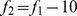 dB SPL) into the ipsilateral ear canal while concurrently recording ear canal sound pressure. The energy of the 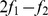 Hz distortion tone, generated by non-linear cochlear amplification of the input tones, was monitored and threshold defined as the 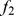 tone intensity which generates a distortion tone greater than 0 dB SPL (noise floor 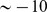 dB SPL).

Differences between mutant and wildtype threshold, magnitude and latency values were tested for statistical significance using Welch's t test. ABR threshold, amplitude and latency data are from N = 7, 3 and 3 animals from the *Fgf* 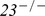, *Fgf* 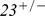, and *Fgf* 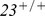 genotypes respectively. DPOAE thresholds were measured from N = 5, 4 and 9 ears of each genotype.

### Histology and Immunohistochemistry

Histologic sections were prepared to assess the microscopic anatomy of the inner ear. Mice were anesthetized using ketamine (0.1 mg/g) and xylazine (0.02 mg/g) before intra-cardiac perfusion. 4% paraformaldehyde (PFA) was used for paraffin embedding and 1.5% PFA/2.5% gluteraldehyde for araldite work. A ventral approach was performed to expose the bulla and tympanic membrane for photography and extraction. The round and oval windows were perforated and intra-cochlear perfusion was performed to improve tissue preservation. Cochleae were extracted and submerged in fixative for 2 hours at room temperature. Samples for paraffin embedding were decalcified in EDTA for 2 days and stained with hematoxylin and eosin. Cochleae for araldite embedding were incubated in 1% osmium tetroxide for 1 hour and decalcified in EDTA with 1% gluteraldehyde for 3–4 days at room temperature.

Slides for FGF23 immunostaining were deparaffinized in xylene and rehydrated with an alcohol ladder (100%, 95%, 75% and 50%). Sections were immersed in 3% 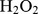 in methanol, then blocked in 5% goat serum for 30 minutes, and incubated with rat anti-FGF23 primary antibody (AMGEN, 1∶200) overnight at 4°C. Biotinylated goat anti-rat secondary antibody (Vector Laboratories, BA-9401, 1∶200) was applied for 1 hour at room temperature followed by HRP substrate (BD Pharmingen, 92121) and developed with DAB for 2 minutes (Vector Laboratories, SK4100, 1∶10). Slides were counterstained with hematoxylin (VWR, 95057-844).

### *μ*CT analysis

Gross morphology of the bulla, ossicles and cochlea of *Fgf* 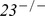 and *Fgf* 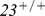 mice were studied using high-voltage X-ray computed tomography (*μ*CT). Mice were anesthetized and intra-cardially fixed as above. Soft tissues were removed from the head to expose the skull. The cranium and hard palate were carefully dissected, leaving only the portion of the skull base between the bullae and extending forward to contain the bony ear-canals. The tympanic membrane of each ear was perforated and samples were fixed overnight in 4% PFA.

*μ*CT scans were performed on a Scanco Medical *μ*CT35 System (Scanco Medical, Brüttlsellen, Switzerland). Scans were conducted in 70% ethanol with parameters: voxel size - 7 *μ*m, X-ray tube potential - 55 kVp, X-ray intensity - 0.145 mA and integration time - 600 ms (as detailed in [31]).

Raw *μ*CT data were imported into Voxar 3D 6.3 (Toshiba Medical Visualization Systems, Japan). For the 2D reformats, the raw data for each ear were separated and then brought up on a clinical imaging work station viewing console, where the raw data were displayed in three orthogonal planes. The data were first reformatted into a dataset of 7 *μ*m thick images rendered in a plane parallel to the posterior semicircular canal (PSCC) and sagittal to the head. An orthogonal coronal data set was then made in a plane perpendicular to the PSCC and coronal to the head. For the 3D reconstructions, the raw data for each ear were displayed using 3D volume rendering with preset window level settings that were optimized for middle ear analysis (settings provided by the software developer). Regions of interest were manually segmented to exclude the walls of the middle ear and to isolate the ossicles.

The sagittal and coronal images, and segmented 3D reconstructions from the *Fgf* 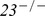 samples were reviewed and compared with the *Fgf* 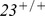 specimens with regard to morphology of the ossicles, contour of the incudomalleal joint, pneumatization of the petrous apex, pneumatization of the mastoid, Eustachian tube patency and CT homogeneity of the bulla, otic capsule and ossicles.

## Results

### Anatomic Findings

**Figure 1:**
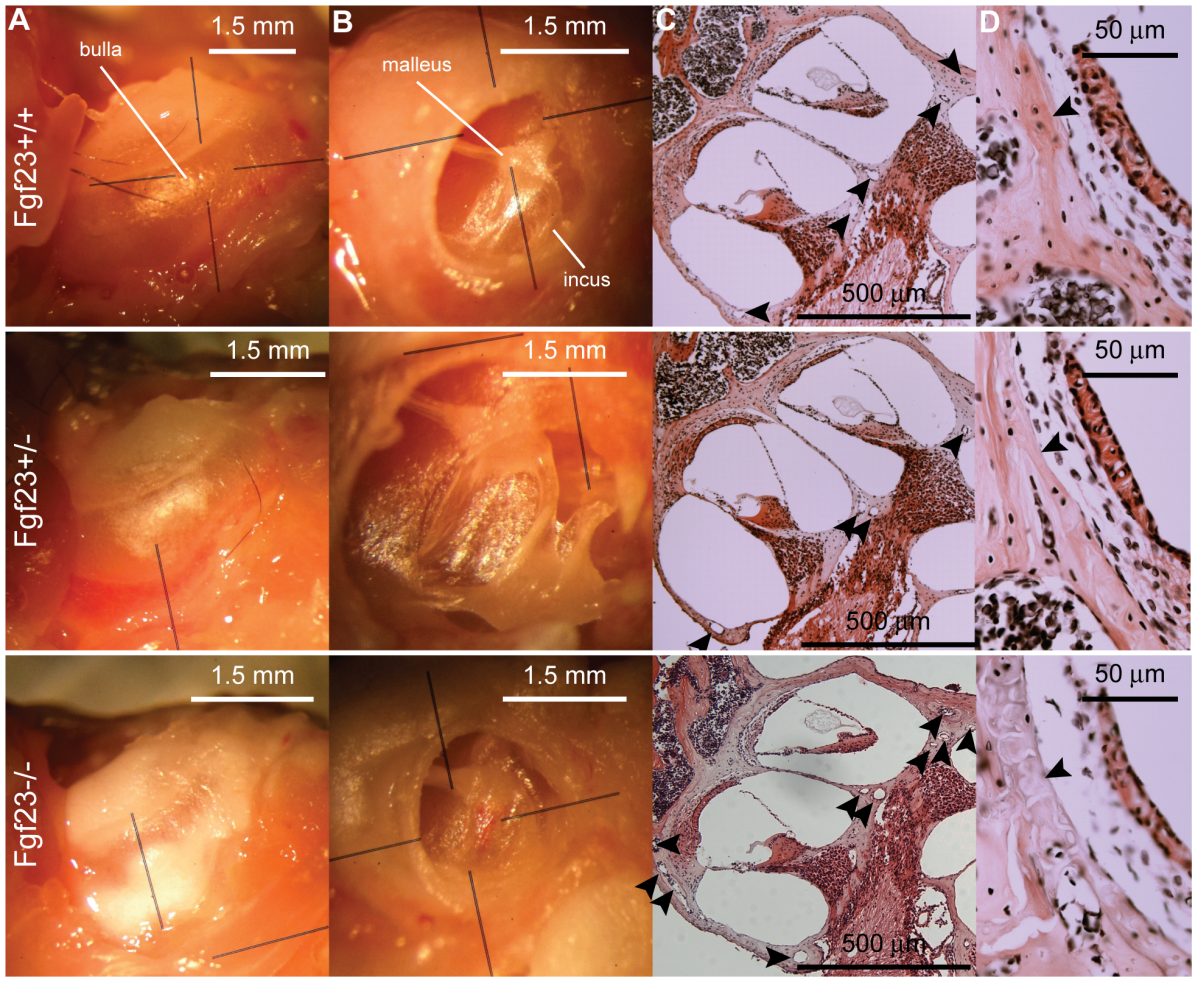
Morphological and histological comparisons of the middle and inner ears. (A) The bullae of *Fgf* 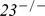 mice (bottom row) appear white and cloudy with less structural refinement than both *Fgf* 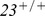 (top row) and *Fgf* 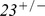 mice (middle row), indicating incomplete ossification. (B) The auditory ossicles are dysplastic in *Fgf* 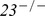 mice. (C) H&E stained, paraffin sections demonstrate increased vascularization of the bony labyrinth in *Fgf* 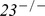 mice (arrowheads). (D) The highly-organized laminar structure of the otic capsule, bordering the spiral ligament, is lost in the *Fgf* 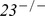 genotype. Lines in A and B are orienting lines from the microscope objective.

**Figure 2:**
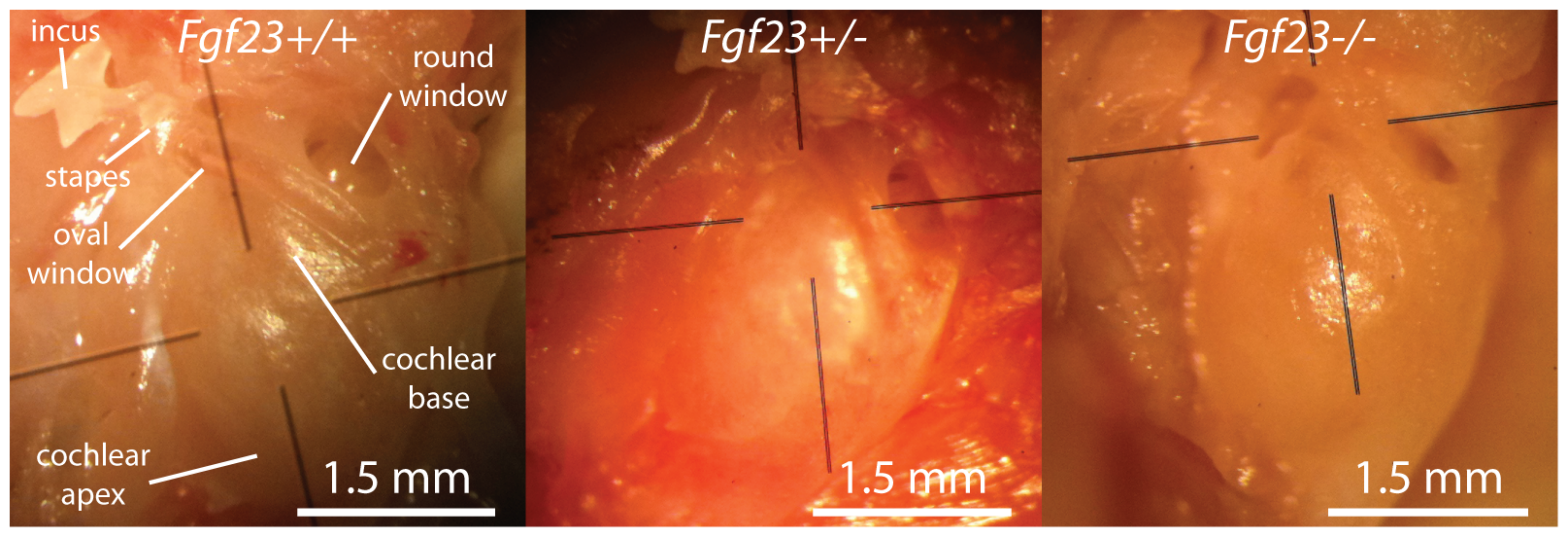
Morphological comparisons of the otic capsule from (A) *Fgf* 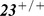, (B) *Fgf* 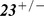 and (C) *Fgf* 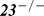 mice. All genotypes appear similar but the *Fgf* 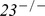 cochlea is slightly smaller and whiter. Black lines are orienting lines from the microscope objective.

Morphological assessments of middle and inner ear anatomy revealed that bullae from *Fgf* 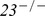 mice appear cloudy and lack the precise refinement in shape that is characteristic in *Fgf* 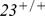 mice (Fig. 1A). The auditory ossicles are similarly a cloudy white hue and show significant dysplasia, consistent with abnormal bone remodeling (Fig. 1B). In contrast, the cochleae and vestibular organs of all genotypes appear similar, although *Fgf* 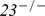 cochleae are slightly smaller and whiter (Fig. 2). The bullae of *Fgf* 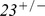 mice have normal shape but small, white, cloudy patches. The ossicles appear slightly dysplastic. In no cases did the *Fgf* 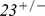 morphological phenotype approach the severity of the *Fgf* 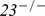 mice. Middle ear effusions and signs of otitis media were observed in 4 of 5 *Fgf* 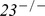 and 1 of 3 *Fgf* 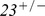 mice. Our finding of severe middle-ear phenotypes in *Fgf* 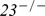 mice are consistent with the phenotype described in other bones, including severe axial and appendicular skeletal malformations, characterized by nodules, rachitic lesions and narrowed growth plates [2]. Others have not observed obvious abnormality in the *Fgf* 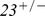 genotype [9].

**Figure 3:**
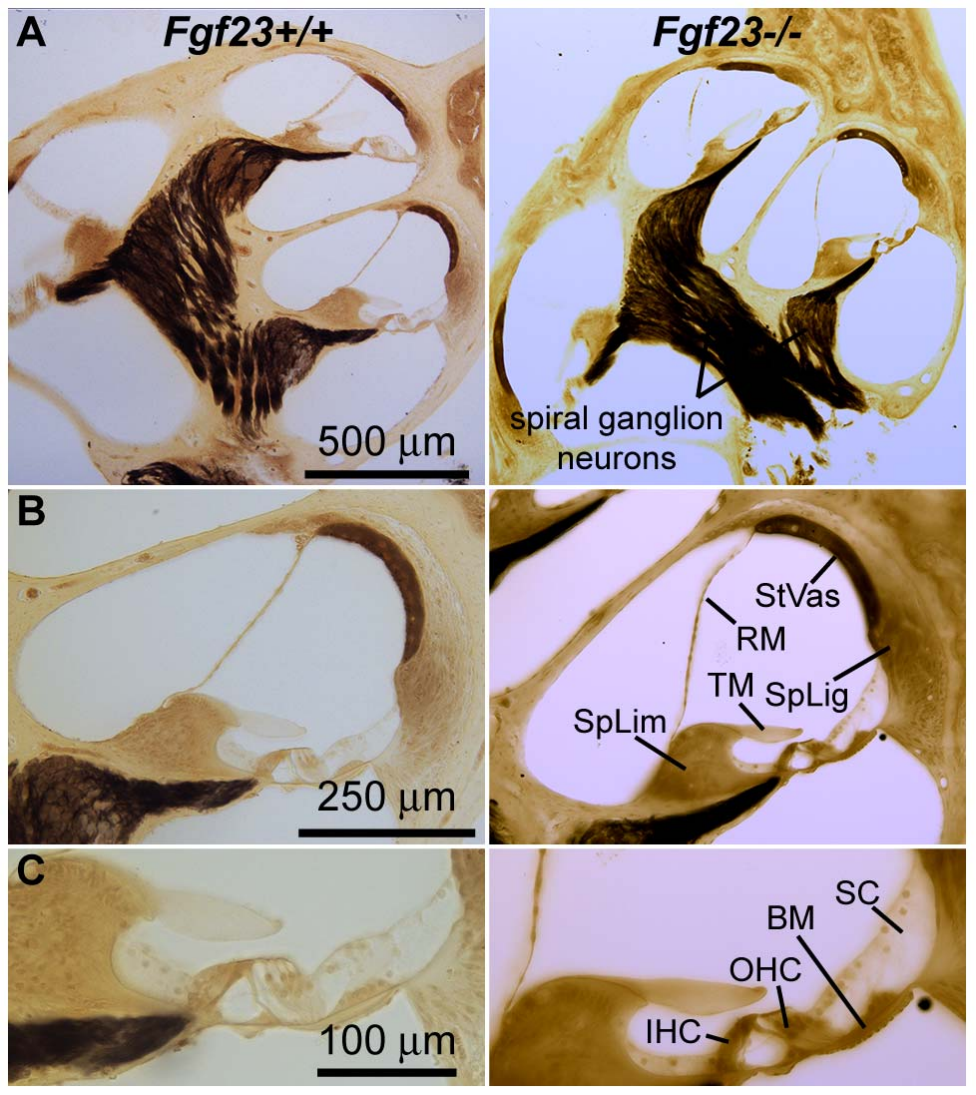
Mid-modiolar cochlear sections from *Fgf* 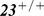 (left column) and *Fgf* 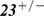 mice (right column). (A) Neural populations and gross anatomical structure appear normal in *Fgf* 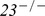 mice. (B) The stria vascularis (StVas), spiral ligament (SpLig), spiral limbus (SpLim), tectorial membrane (TM) and Reissner's membrane (RM) are similar to *Fgf* 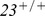 mice. (C) Inner hair cells (IHC), Outer hair cells (OHC), supporting cells (SC) and basilar membrane (BM) are morphologically normal. Sections were embedded in araldite and osmium stained.

The membranous labyrinth of the inner ear appears anatomically normal, healthy and properly organized in *Fgf* 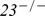 mice (Fig. 3). However, inspection of the bony labyrinth in *Fgf* 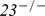 mice uncovers phenotypic differences. The modiolus appears to have increased vascularization (Fig. 1C, arrowheads) and the highly organized laminar sheets of the otic capsule are replaced by swirling structures in the region bordering the spiral ligament (Fig. 1D).

**Figure 4:**
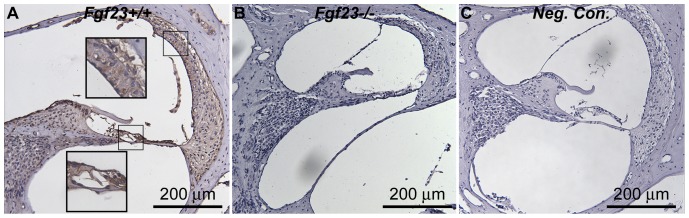
FGF23 immunohistochemistry in *Fgf* 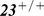 and *Fgf* 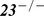 mice. (A) Specific staining was observed in the cells of the spiral ligament, stria vascularis, Organ of Corti, spiral limbus and within the spiral ganglion neuronal cell bodies in *Fgf* 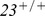 mice (similar patterns were observed in *Fgf* 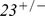 mice). Inset images are zoomed in views of the boxed regions. (B) No immunoreactivity was observed in *Fgf* 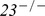 mice. (C) Similarly, no staining was observed in negative controls, from *Fgf* 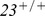 mice which were processed without the primary antibody.

Immunostaining of cochlear slides revealed the widespread presence of FGF23 throughout most cell types of the inner ear (Fig. 4). Immunoreactivity was observed within cells of the spiral ligament, stria vascularis, Organ of Corti, spiral limbus and within the spiral ganglion neuronal cell bodies as well as osteocytes in *Fgf* 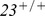 (Fig. 4A) and *Fgf* 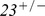 mice (not shown). The strongest staining was observed in the stria vascularis and Organ of Corti. No Immunoreactivity was observed in *Fgf* 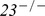 mice (Fig. 4B), or in *Fgf* 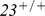 mice processed without the primary antibody (Fig. 4C), indicating the antibodies are reacting specifically.

### *μ*CT Reconstruction

**Figure 5:**
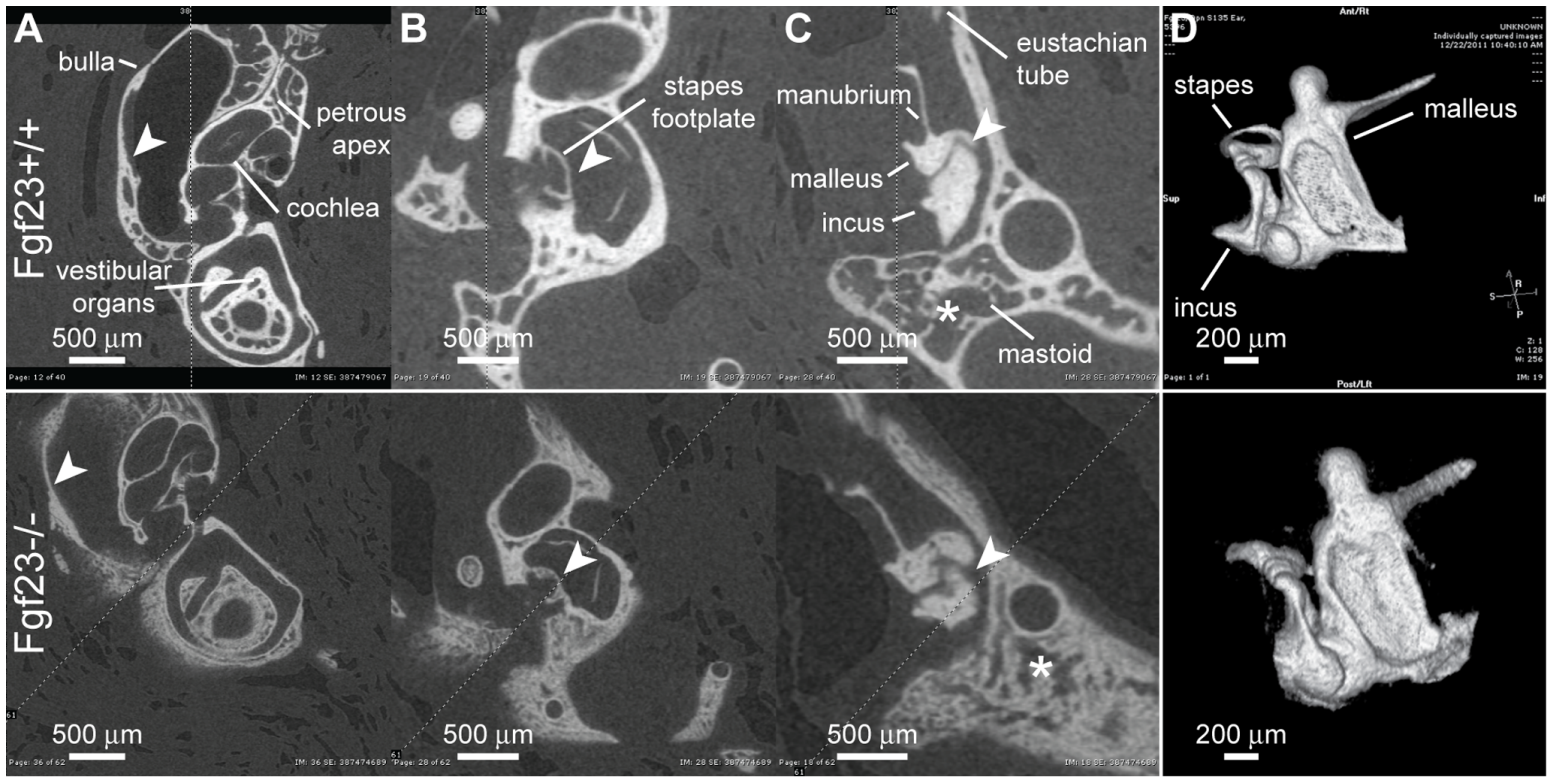
2D and 3D 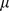
CT reconstructions of bullae from *Fgf* 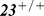 and *Fgf* 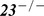 mice. Each row contains reconstructions from one ear (top row: *Fgf* 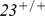, bottom row: *Fgf* 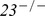). (A) The otic capsule and bulla show loss of structural refinement and decreased density (arrowheads). (B) In *Fgf* 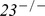 mice the footplate of the stapes demonstrate thickening (arrowheads). (C) The incus and incudomalleal joint are dysplastic (arrowheads) and the mastoid is under-pneumatized (asterisks). (D) The borders of 3D reconstructed *Fgf* 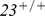 ossicles are sharp and well-defined while those of *Fgf* 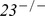 ossicles are blurry due to poor contrast with surroundings, resulting from decreased bone density in the mutant. *Fgf* 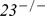 ossicles demonstrate reduced structural refinement, particularly in the malleus and incus.

Comparison of 2D *µ*CT sections from *Fgf* 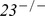 and *Fgf* 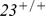 mice (N = 2,2) uncovered several bony phenotypes of the ossicles and mastoid bone. The malleal head appears dysplastic in *Fgf* 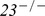 specimens (Fig. 5C), as does the incus, either abnormally notched or heart-shaped, and the stapes demonstrates thickening of the crua and footplate (Fig. 5B). The incudomalleal joint appears abnormally contoured in the *Fgf* 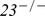 genotype, rough, irregular and notched, compared to the smoothly contoured, tight fitting and angular interface of the *Fgf* 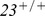 mice (Fig. 5C). The mastoid (Fig. 5C) and petrous apex (Fig. 5B) are substantially under-pneumatized. The bony portion of the Eustachian tube is patent and similar to *Fgf* 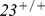 mice (Fig. 5C). The incus and malleal head appear heterogeneously lucent when compared to the homogeneously dense formations found in the *Fgf* 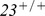 mice (Fig. 5C). Similarly, the bulla, otic capsule and vestibular compartments are characterized by lower density and poor lamellar organization (Fig. 5A).

Three-dimensional reconstruction of the ossicles highlights the lack of structural refinement in *Fgf* 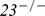 mice (Fig. 5D). Regions with particularly noticeable dysplasias include the incudal pocket, manubrium and separation of the malleal ridge and head. The lucency of the *Fgf* 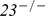 bones, versus the diffusely dense *Fgf* 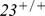 counterparts (noted above), is reflected in the unsharpness of the borders of both 3D reconstructed *Fgf* 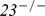 ossicular chains. This results from poor *µ*CT contrast with surroundings due to lower ossicular density.

### Audiometric Assessment

**Figure 6:**
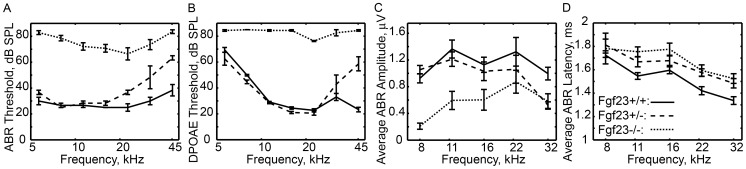
Auditory measurements from *Fgf* mutant mice. (A) ABR and (B) DPOAE thresholds demonstrate profound hearing loss at all frequencies in *Fgf* 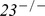 mice, and moderate hearing loss at high frequencies in *Fgf* 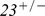 mice. (C) ABR wave I amplitudes appear slightly depressed in *Fgf* 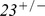 mice but are not statistically differentiable (except at 32 kHz where threshold differences impact magnitude 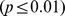). ABR amplitudes in *Fgf* 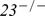 mice are significantly reduced compared to the other genotypes 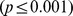. (D) Threshold adjusted ABR wave I latency measurements demonstrate significant increases in latency in both *Fgf* 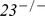 and *Fgf* 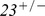 genotypes when compared to *Fgf* 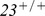 littermates 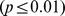. Error bars present standard error of the mean.

ABR measurements (Fig. 6A) demonstrate that *Fgf* 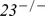 mice have profound hearing loss across all frequencies compared to *Fgf* 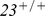 littermates (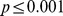). *Fgf* 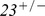 mice have normal hearing below 20 kHz and losses of up to 25 dB above that frequency. DPOAE measurements demonstrate similar trends (Fig. 6B): *Fgf* 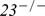 mice have nearly complete hearing loss while *Fgf* 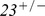 mice demonstrate 40 dB of loss at the highest frequencies and have normal hearing below 30 kHz.

Because ABR *threshold* measurements are known to be insensitive to certain types of neural damage [32], ABR wave I *amplitude* and *latency* were also analyzed. Changes in the amplitude of ABR wave I can be more sensitive than threshold measurements to certain forms of auditory pathology and signal neuronal malfunction months before histological observation of neuronal loss [32]. Figure 6C presents the peak-to-peak amplitude of ABR wave I as a function of tone frequency. For each frequency and genotype, the data represents the average amplitude of response to the 60, 70 and 80 dB SPL tone presentations. Wave I amplitudes are statistically similar between the *Fgf* 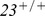 and *Fgf* 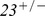 groups at all frequencies except 32 kHz. At this frequency, the 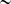
20 dB loss detected by threshold measurements (Fig. 6A) is affecting a significant reduction in amplitude 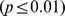. ABRs were only consistently measured from *Fgf* 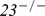 mice at 80 dB SPL for each frequency (due to extremely high thresholds) and were significantly reduced across the frequency range 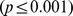.

*Threshold-adjusted* ABR wave I latency values are presented in Figure 6D. Unlike amplitude, it is customary to present latency values for tone levels relative to each animal's hearing threshold (dB SL) rather than absolute level (dB SPL) [33]. For each frequency, the data represent the average response latency to tones presented at 5, 10 and 15 dB SL. Both *Fgf* 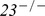 and *Fgf* 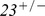 mice have increased wave I latencies, indicating slower neural signaling. The observed increase is most significant at high frequencies and more pronounced in the *Fgf* 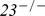 genotype.

These results demonstrate profound hearing loss in *Fgf* 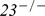 mice, resulting in large part from conductive deficiencies due to ossicular dysplasia. *Fgf* 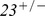 mice demonstrate high-frequency hearing loss, of mixed conductive and sensorineural origin. Significant DPOAE threshold shifts at high-frequencies provide evidence for a conductive component to the loss, while the lower onset frequency of the ABR threshold shifts indicates a sensorineural component. ABR wave I latency data also support the presence of a sensorineural pathology. The near equivalence of ABR amplitudes in *Fgf* 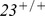 and *Fgf* 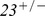 mice suggests that the functional neural populations are of similar size (in agreement with histology), but the increased latency in *Fgf* 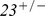 mice indicates a retrocochlear phenotype as a result of the mutation [33]. The increased latency is particularly interesting as no previous study has identified a heterozygote phenotype.

## Discussion

Our findings of significant conductive impairments in *Fgf* 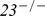 mice coupled with a sensorineural component indicate that the mechanisms of hearing loss due to FGF23 and KL deficiency are different, albeit possibly overlapping. KL expression has previously been demonstrated in the cochlea, specifically in the stria vascularis, spiral ligament, outer hair cells, inner hair cells and spiral ganglion cells [22], [23], potentially enabling tissue specific FGF23 sensitivity. Our immunostaining results demonstrate that FGF23 is even more widely present throughout the ear than KL, and suggest that FGF23-KL interaction may be occurring in many of the inner ear's critical tissues. *Kl* ^−/−^ mice have been shown to exhibit hearing loss; 14–18 dB threshold shifts have been detected in ABRs elicited by clicks as well as tones at 4, 8, 16 and 32 kHz in 3 week old 129/SvJ mice [21]. Another study, more directly comparable to our study due to the common background strain and similar animal age, observed an 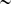
18 dB shift in ABR thresholds for 8 kHz tones in 6–14 week old C57/Bl6 mice [22]. The authors also analyzed *non-threshold adjusted* ABR wave I latency in response to 100 dB SPL tones and found a significant delay of 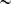
0.4 ms in *Kl* ^−/−^ mice.

The 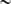
50 dB ABR threshold shift we observed across all frequencies in *Fgf* 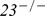 mice is much more severe than the *Kl* ^−/−^ phenotypes. Conversely, the observed ABR wave I latency delays were more severe in *Kl* ^−/−^ mice than the 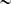
0.2 ms delays at higher frequencies in *Fgf* 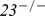 mice (we observed no significant delay at 8 kHz). However, because the results from *Kl* ^−/−^ mice were non-threshold adjusted, these metrics are not directly comparable. ABR latency decreases as tone intensity (in dB SL) increases; therefore the latency difference observed in *Kl* ^−/−^ mice may have a significant contribution owing to the 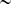
18 dB threshold shift (the significance of this effect is reduced because tones are well above threshold for both groups). The threshold-adjusted metric we utilized, where ABR latencies are measured just above animal threshold, prevents sensitivity differences from influencing the latency and allows the result to be more demonstrative of actual differences in the rate of signal transduction and neural conduction.

Unfortunately, neither of the above studies of *Klotho* mutations presented hearing results from *Kl^+/−^* mice; thus comparison with the unexpected *Fgf* 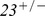 phenotype is not possible. However, the mixed hearing loss associated with the *Fgf* 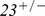 phenotype is intriguing, particularly the presence of significant threshold-adjusted latency delays at frequencies below DPOAE threshold shifts, strongly indicating a neural phenotype. Overall, this divergence of *Fgf* 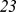 and *Kl* phenotypes is made more interesting by recent research which has suggested a calcineurin-mediated FGF23 signaling pathway [28], and activation of calcineurin has previously been shown to contribute to noise induced hearing loss [34], [35].

Our results raise questions concerning the prevalence of hearing loss among individuals with FGF23 deficiencies. No auditory phenotype has been reported in FGF23-mediated familial tumoral calcinosis, which results from a missense mutation in *FGF23*. However, diseases with known elevations in serum concentrations of FGF23 co-present with hearing loss [36]. Our findings demonstrate that FGF23 deficiency is sufficient to induce profound sensory impairment in mice. The lack of such an obvious phenotype in humans suggests important differences between species, potentially stemming from robust protection against hypervitaminosis D in humans [21]. Alternatively, a putative auditory phenotype in humans may be less severe, and therefore less studied, than the debilitating systemic phenotypes.

Our findings also suggest a potential role for FGF23 in otitis media, a clinically important disease that afflicts 75–90% of Americans at least once before 3 years of age [37], [38]. Otitis media is estimated to cost the national healthcare system more than $4 billion annually [30], and in extreme cases can result in long-term speech and learning impairments [38]. Currently, genetic risk factors are poorly understood [29], but the prevalence of pediatric cases is commonly believed to be related to the ongoing maturation of the Eustachian tube [39], which undergoes significant development in the first decade of life. Interestingly, hyperphosphatemia in *Fgf* 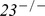 mice (which precedes other observed symptoms) first appears at P10 [9]. It is believed that a FGF23-independent mechanism of phosphate homeostasis must be functioning prior to this age. Our observations support this suggestion as structures which are nearly mature by P10 show very little dysplasia while structures undergoing continued maturation demonstrate more drastic phenotypes. At P10, the ossicles of wildtype mice are still surrounded by mesenchyme, indicating incomplete development, while the remainder of the inner ear space has begun to clear the mesenchymal tissue [40]. In humans with reduced FGF23 activity, familial tumoral calcinosis, hyperphosphatemia may present as early as 21 months old [41], indicating that FGF23 is important in phosphate handling when most episodes of otitis media develop. Given the potential temporal and mechanistic overlap of initiation of FGF23 activity and Eustachian tube development, this work suggests a possible role for FGF23 in the genetic predisposition to otitis media, a link that warrants further investigation.
